# Construction of a miRNA-Based Nomogram Model to Predict the Prognosis of Endometrial Cancer

**DOI:** 10.3390/jpm12071154

**Published:** 2022-07-17

**Authors:** Leyi Ni, Chengyun Tang, Yuning Wang, Jiaming Wan, Morgan G. Charles, Zilong Zhang, Chen Li, Ruijie Zeng, Yiyao Jin, Penghao Song, Ming Wei, Bocen Li, Jin Zhang, Zhenghao Wu

**Affiliations:** 1 School of Medicine, I.M. Sechenov First Moscow State Medical University, Ministry of Health, Russian Federation, 8/2 Trubetskaya Street, 119991 Moscow, Russia; nly261262@outlook.com (L.N.); tangchengyun2014@hotmail.com (C.T.); ennewing00@outlook.com (Y.W.); wan13966503316@gmail.com (J.W.); justgilbert223@gmail.com (M.G.C.); zhangzilong6722@gmail.com (Z.Z.); jyy727826@gmail.com (R.Z.); jinyiyao111@gmail.com (Y.J.); songpenghao135@gmail.com (P.S.); weiming9872@gmail.com (M.W.); libocen0101@gmail.com (B.L.); 2 Department of Biology, Chemistry, Pharmacy, Free University of Berlin, 14195 Berlin, Germany; chen.li@fu-berlin.de; 3 Department of Pharmacology and Toxicology, University of Mississippi Medical Center, Jackson, MS 39216, USA

**Keywords:** endometrial cancer, differentially expressed microRNA, TCGA, nomogram, survival analysis

## Abstract

**Objective**: To investigate the differential expression of microRNA (miRNA) in patients with endometrial cancer and its relationship with prognosis and survival. **Method**: We used The Cancer Genome Atlas (TCGA) database to analyze differentially expressed miRNAs in endometrial cancer tissues and adjacent normal tissues. In addition, we successfully screened out key microRNAs to build nomogram models for predicting prognosis and we performed survival analysis on the key miRNAs as well. **Result**: We identified 187 differentially expressed miRNAs, which includes 134 up-regulated miRNAs and 53 down-regulated miRNAs. Further univariate Cox regression analysis screened out 47 significantly differentially expressed miRNAs and selected 12 miRNAs from which the prognostic nomogram model for ECA patients by LASSO analysis was constructed. Survival analysis showed that high expression of hsa-mir-138-2, hsa-mir-548f-1, hsa-mir-934, hsa-mir-940, and hsa-mir-4758 as well as low-expression of hsa-mir-146a, hsa-mir-3170, hsa-mir-3614, hsa-mir-3616, and hsa-mir-4687 are associated with poor prognosis in EC patients. However, significant correlations between the expressions levels of has-mir-876 and hsa-mir-1269a and patients’ prognosis are not found. **Conclusion**: Our study found that 12 significantly differentially expressed miRNAs might promote the proliferation, invasion, and metastasis of cancer cells by regulating the expression of upstream target genes, thereby affecting the prognosis of patients with endometrial cancer.

## 1. Introduction

Endometrial cancer (EC), is the sixth most commonly diagnosed cancer and the 14th leading cause of cancer death in women worldwide [1], and its incidence is increasing globally with changes in lifestyle risk factors including increasing obesity and diet and hormone replacement therapy use [2]. The mean age at diagnosis is 61 years old [3]. Postmenopausal bleeding is the leading symptom in patients with EC [4,5]. This urges patients to seek medical evaluation, and that is why the mortality rate is much lower than other gynecologic cancers [6]. For asymptomatic patients, screening is recommended only for those with hereditary nonpolyposis colorectal cancer [7]. According to histopathological features, EC can be classified into two types, which carries significant prognostic difference. EC-type 1 (endometrioid adenocarcinoma grade 1 and 2) is associated with better prognosis because the adenocarcinoma occurs from atypical endometrial hyperplasia, which is associated with long-term unopposed estrogen stimulation relative to progesterone, while EC-type 2 (endometrioid adenocarcinoma grade 3 or non-endometrioid histology) is hormone-independent. This is more likely to relapse and metastasize [8]. Type 1 carcinoma has been identified to have association with PTEN inactivation, PI3KCA upregulation, K-ras mutation, β-catenin mutation, and microsatellite instability [9,10]. However, p53 mutation, HER-2/neu overexpression, P16 inactivation, and E-cadherin reduction contribute mainly to type 2 EC [11]. Total hysterectomy with bilateral salpingo-oophorectomy with or without lymphadenectomy is the mainstay of treatment. In addition, systemic chemotherapy and radiotherapy can be added but their benefits for improving overall prognosis still have to be elucidated [12]. Despite the fact that prognostic factors for EC such as clinical, surgical, and histopathological have been well assessed, the discovery of a genetic and molecular marker is required to better classify EC and evaluate prognosis [13].

Through a comprehensive proteogenomic characterization of endometrial carcinomas, the researchers identified microRNA associations of potential mechanistic significance in the development of endometrial cancers [14]. MicroRNAs (miRNAs) are small non-coding RNAs with a length of about 19–25 nt [15], which can regulate various target genes in numerous biological processes and diseases [16,17,18] including cancer initiation and progression through amplification, deletion, mutation, and epigenetic silencing [12,19]. Consequently, studying EC-associated microRNAs offers an encouraging prospective in assessing EC diagnosis and prognosis. The Cancer Genome Atlas (TCGA) [20] is a publicly funded project that provides a comprehensive cancer genome atlas that allows researchers to explore cancer-related knowledge, helps us understand cancer pathogenesis, and facilitates diagnosis or therapeutic intervention. Based on this database, we studied the expression levels of prognosis-related miRNAs in EC patients and built a model for predicting the survival rate of EC patients. We established a nomogram based on 12 miRNAs, and subsequently validated their specificity in EC patients. This finding may help us to refine current diagnosis, treatment, and prognosis prediction in patients with EC.

## 2. Materials and Methods

### 2.1. Data Resources and Preprocessing

In our study, patients’ miRNA differential expression data and clinical information were obtained from the TCGA data portal (http://tcga.cancer.gov) (accessed on 23 April 2022) [21]. miRNA expression data were obtained from a total of 569 specimens, including 536 EC specimens and 33 non-tumor tissues. The “edgeR” package in the R software was used to screen and identify differentially expressed miRNAs between tumor tissue and adjacent nontumor tissue, with a threshold of |log 2 FG| > 2 and an adjusted *p* value (FDR) < 0.05 [22]. This study complies with TCGA’s data access policy. We exempted relevant ethnical approval from these studies because the TCGA database is public.

### 2.2. Identification of Potential Prognostic miRNAs

To assess the prognostic value of these miRNAs, we used Cox regression analysis to identify miRNAs that were significantly associated with EC. We conducted Univariate Cox analysis to determine the association between miRNA expression levels and patient survival, with statistical significance set at *p* < 0.05. Statistically significant miRNAs were further analyzed using least absolute shrinkage and selection operator (LASSO) univariate analysis. miRNAs with *p* values < 0.05 were further subjected to multivariate Cox regression analysis to determine predictive miRNAs.

### 2.3. Establishment of EC Prognostic Risk Model

The dataset (*n* = 536) was used to develop a clinical prediction model. Half of the dataset (*n* = 268) was randomly selected as a validation dataset for validating the accuracy of the prognostic model based on miRNAs as predictors for EC patients. LASSO regression comes with a cross-validation model that selects variables with different underlying parameters to build the best model. In the process, LASSO automatically helps to eliminate or distort highly correlated and redundant features with a low variance method. Subsequently, the “rms” R package was used to analyze the significantly differentially expressed miRNA variables screened by LASSO analysis, and to predict the 3-year and 5-year survival rate by nomogram, and establish a nomogram model for predicting the prognosis of EC.

### 2.4. Calibration and Validation of the EC Survival Nomogram Model (Predictions)

Calibration curves were built using the “rms” and “survival” R packages to correct for nomogram-predicted 3- and 5-year survival and to evaluate the agreement between the prediction model results and actual clinical outcomes. We constructed receiver operating characteristic (ROC) curves using the “ROC” R package to determine the specificity and sensitivity of prognostic miRNA risk models.

### 2.5. Visualization of Risk Score of EC Patients in High- and Low-Risk Groups

We built a risk scoring formula based on its Cox coefficient.

Specific risk score formula:

Risk score = ∑Coef miRNA × log2 (miRNA expression + 1).

Based on the median risk score, TCGA EC patients were divided into high-risk and low-risk groups. The risk scores of EC patient (high-risk and low-risk) groups are seen using the “ggplot2”, and R package and survival curves were constructed.

### 2.6. Survival Analysis of Differentially Expressed miRNAs

We performed survival analysis on the screened differentially expressed miRNAs using the “survival” R package. We made calculations of Hazard ratios (HRs) with 95 CIs and log-rank *p*-values to assess the prognostic value of miRNAs.

### 2.7. Statistical Analyses

We performed all statistical analysis in this study using R software (version 3.6.2). All statistical tests were two-sided and adjusted *p* < 0.05 for statistical significance.

## 3. Results

### 3.1. Determining the Significance of Differential Expression of miRNAs

**Figure 1:**
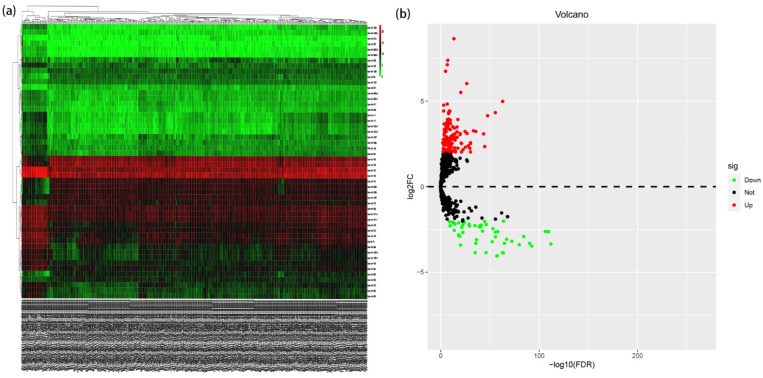
Identification of DEMs associated with EC patients: (**a**) the heatmap of 50 DEMs. Colors represent the expression level of the genes, with darker colors indicating higher expression; red, upregulated; green, downregulated. (**b**) miRNAs vocano plot. A total of 679 miRNAs were identified with the thresholds as |log2foldchange| > 2 and *p* value < 0.05.

**Figure 2:**
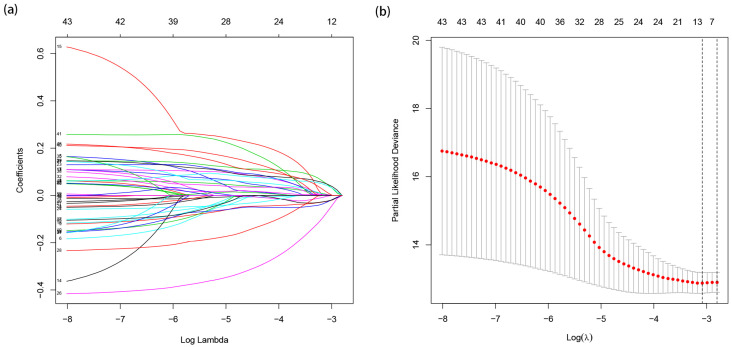
(**a**) Distribution of LASSO coefficients for 47 related miRNAs. (**b**) Partial likelihood bias of the LASSO coefficient distribution. The vertical dashed line indicates the minimum partial likelihood deviation.

**Figure 3:**
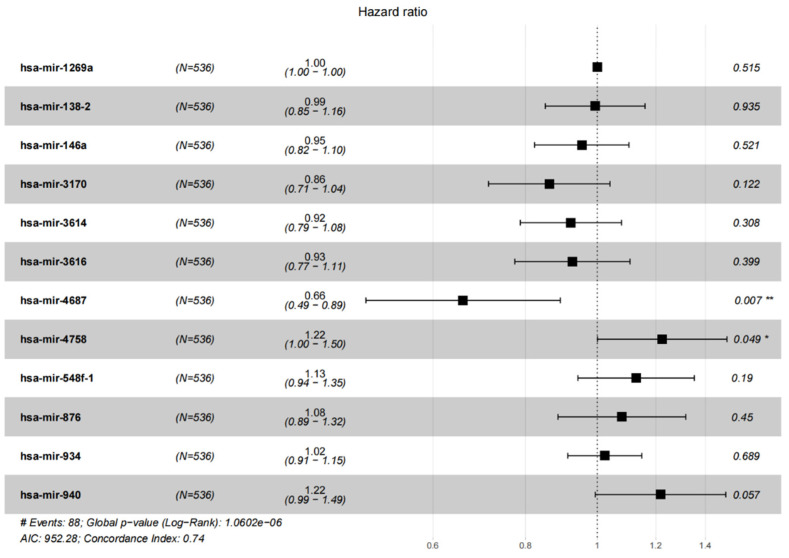
Multivariate COX analysis was used to verify 12 miRNAs related to EC patients. *: *p* < 0.05; **: *p* < 0.01.

A total of 187 differentially expressed miRNAs were screened, including 134 up-regulated differentially expressed miRNAs and 53 down-regulated differentially expressed miRNAs, Figure 1a,b. Univariate Cox regression analysis identified 47 miRNAs which are significantly associated with EC. Through LASSO analysis, finally 12 miRNAs significantly associated with the prognosis were retained, Figure 2a,b. The regression coefficients of 12 miRNAs which were significantly related to prognosis were further determined by multivariate Cox regression analysis, and a forest plot was established, Figure 3. C-index = 0.74 indicates that the selected miRNA variables have better accuracy for predicting the survival for EC patient.

### 3.2. Construction of EC Prognosis Survival Nomogram Model

**Figure 4:**
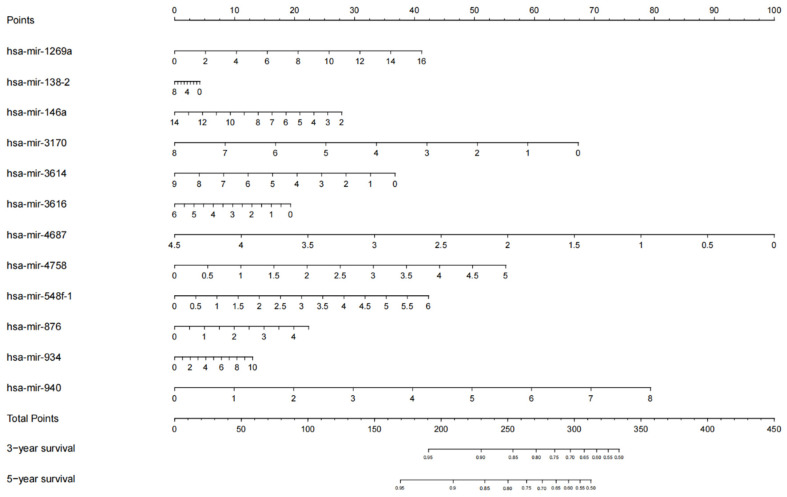
The prognostic nomogram established by 12 miRNAs was used to predict 3- and 5-year OS of patients with EC. Including data derived from 536 EC specimens and 33 non-tumor tissues. The nomograms are interpreted by adding up the points assigned to each variable, as indicated at the top of the point scale. The total point projected on the bottom scale represents the probability of 3- or 5-year OS.

Using the 12 miRNAs screened out by LASSO regression that were significantly associated with prognosis as independent predictors, a nomogram model for predicting EC prognosis was constructed. The prediction of 3-year and 5-year survival probability is shown in Figure 4.

### 3.3. Calibration and Verification of the Nomogram

**Figure 5:**
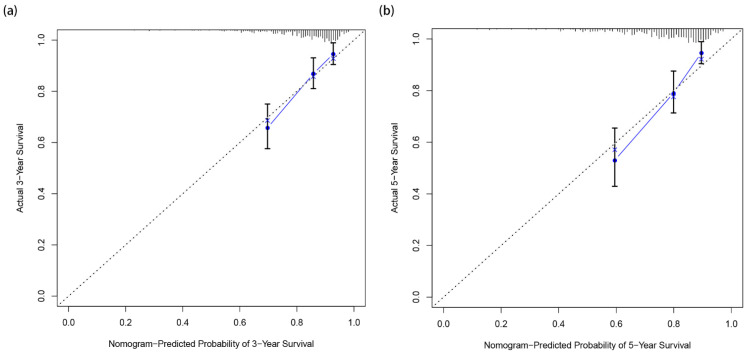
(**a**) Calibration curves of EC patient risk signature used for evaluating the 3- year AUC. (**b**) Calibration curves of EC patient risk signature used for evaluating the b- year AUC.The x-axis is nomogram-predicted probability of survival, and the y-axis is actual survival. We used the bootstrapping method for the internal validation of the nomogram. The dotted line indicates perfect calibration, the blue line represents the actual predictive power of the model, the closer the blue line is to the dotted line, the higher the accuracy of the prediction.

**Figure 6:**
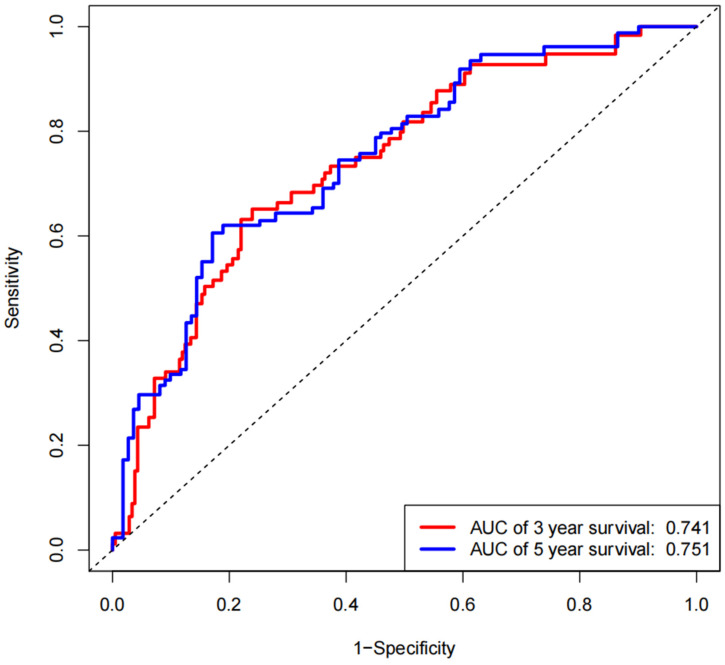
AUC of risk signature in ROC analysis was calculated for EC patients with 3- and 5-year survival time.

Calibration curves for 3-year and 5-year survival probabilities shows that predictions using the nomogram model are in good agreement with those observed in the actual validation cohort, Figure 5a,b. By drawing the ROC curve, the AUC (Area Under Curve) values of EC patients were higher at 3 years (0.741) and 5 years (0.751), and the model had better accuracy in predicting the survival rate, Figure 6. These results suggest that the nomogram has a good ability to predict survival.

### 3.4. Visualization of Risk Score of EC Patients in High- and Low-Risk Groups and Survival Analysis of 12 miRNAs

**Figure 7:**
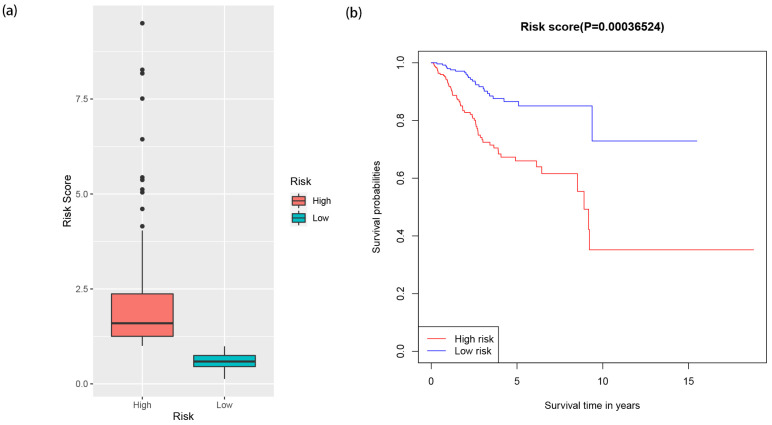
Risk scores of EC patients in high- and low-risk groups. (**a**) The risk scores of EC patients in the high- and low-risk groups displayed by the boxplot. (**b**) Survival analysis of EC patients in high- and low-risk groups.

**Figure 8:**
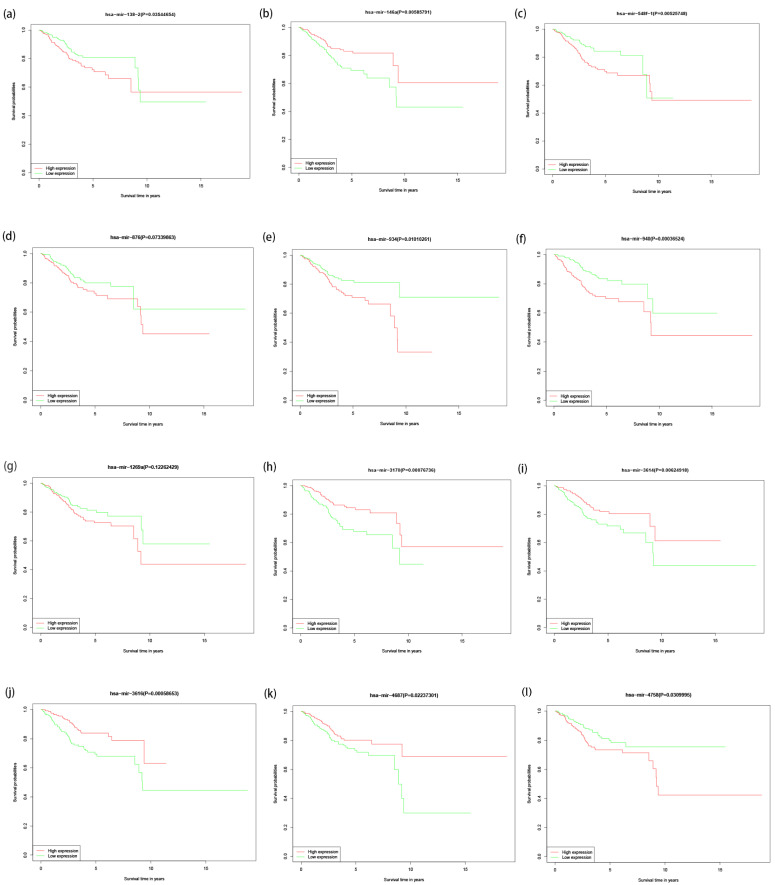
Survival analysis for EC patients with high and low expression of 12 miRNAs: (**a**) hsa-mir-138-2; (**b**) hsa-mir-146a; (**c**) hsa-mir-548f-1; (**d**) hsa-mir-876; (**e**) hsa-mir-934; (**f**) hsa-mir-940; (**g**) hsa-mir-1269a; (**h**) hsa-mir-3170; (**i**) hsa-mir-3641; (**j**) hsa-mir-3616; (**k**) hsa-mir-4687; (**l**) hsa-mir-4758.

The visualization of the risk scores of EC patients in high- and low-risk groups is shown in Figure 7 (*p* < 0.001). In order to verify the expression of 12 miRNAs in paratumor tissues and EC tissues, survival analysis showed that five miRNAs were highly expressed in EC tissues, including hsa-mir-138-2, hsa-mir-548f-1, hsa-mir -934, hsa-mir-940, and hsa-mir-4758. In the meantime, the study also found five miRNAs that are low-expressed in EC tissues, these include: hsa-mir-146a, hsa-mir-3170, hsa-mir-3614, hsa-mir-3616, and hsa-mir-4687. The difference was statistically significant (*p* < 0.05), and there was no significant difference in the expression of hsa-mir-876 and hsa-mir-1269a between EC tissues and adjacent non-tumor tissues, Figure 8.

## 4. Discussion

Endometrial cancer is one of the most common gynecological cancers [23]. The incidence of endometrial cancer is increasing and most patients diagnosed with early or type 1 EC have a good prognosis only after surgery [24]. However, for patients with advanced, metastatic, or type 2 EC, the current approaches are limited to hormonal therapy or cytotoxic chemotherapy and the overall chance of survival remains poor [25]. This suggests that type 1 and 2 represent distinct disease entities and require different treatment modalities. Therefore, understanding key risk factors and their roles in tumorigenesis is crucial for developing such preventive strategies and evaluating prognosis [26].

MicroRNAs (miRNAs), small noncoding 19–25 nucleotides long RNAs, coordinate multiple biological processes and diseases including cancer initiation and progression [11,27,28]. The studies have shown that miRNAs not only control mRNA expression but also target long non-coding RNAs and miRNAs. This is a way of self-regulating [29]. In recent years, abnormal expression of miRNAs has been reported to be associated with the occurrence of various cancers [30,31,32,33,34], and aberrant expression of miRNAs can also induce different epigenetic changes [35]. Falzone et al. found that seven miRNAs were associated with the prognosis of breast cancer [36], which suggests that miRNAs may play a role in the development of breast cancer and may play a role in receiving comprehensive interventions including diet and physical activity. The study by Chen et al. found eight miRNAs associated with colon cancer prognosis by analyzing differentially expressed miRNAs [37]. Liu et al.’s study also found seven miRNAs associated with gastric cancer. This can be used as a potential biomarker for early diagnosis of gastric cancer in patients [38] because abnormally expressed miRNAs can also form a miRNA–mRNA regulatory axis with abnormally expressed mRNAs, which in turn becomes the pathophysiology of HBV-related hepatocellular carcinoma mechanisms to inform the development of new therapeutics [39]. miRNA dysregulation had been confirmed to be related to the occurrence of endometrial cancer, such as by Wang et al. The Wang et al. miRNA expression profile, a total of nine diagnostic miRNA markers, were identified to have a distinctive EC from normal endometrial tissue [40]. Dou et al. found novel molecular associations with potential mechanistic implications [14], while Lu et al. culminated the use of three prognostic miRNAs to construct a prognostic risk signature that correlates with the expression of multiple immune checkpoints. This involved the infiltration of multiple immune cells [41]. However, although some studies have analyzed endometrial cancer-related miRNAs (DOI: 10.1080/21655979.2021.1947940, DOI: 10.1016/j.gene.2019.01.046), our research screened the miRNA data of the latest endometrial cancer samples in TCGA, and finally screened out 12 significantly differentially expressed miRNAs, including those not found in previous studies. We established a list of 12 key miRNAs nomogram model and used it to evaluate the prognosis of EC patients to make prediction results more accurate.

Based on the TCGA database, we analyzed the differentially expressed genes between EC tissue and normal paracancerous tissue, and applied LASSO analysis to help better predict EC patients. Finally, we screened out 12 miRNA variables that can be included in the prediction model, including hsa-mir-138-2, hsa-mir-548f-1, hsa-mir-934, hsa-mir-940, hsa-mir-4758, hsa-mir-146a, hsa-mir-3170, hsa-mir-3614, hsa-mir-3616, hsa-mir-4687, hsa-mir-876, and hsa-mir-1269a. hsa-mir-138-2 is from the MIR138 family of microRNAs. High expression of hsa-mir-138 has been identified as an important prognostic factor in cervical cancer [42] and as a useful tool for the detection of recurrence in ovarian clear cell carcinoma. Serum biomarkers [43], similar to the results of this study, had high expression of hsa-mir-138 which can down-regulate cell adhesion. It may be associated with tumor cell metastasis and have an impact on prognosis. Abnormal expression of upstream gene BRCA may also cause high expression of the hsa-mir-548 binding site; this has been confirmed to be associated with the pathogenesis of hereditary breast and ovarian cancer [44]. The results of this study found that high expression of hsa-mir-548 has a negative correlation with the prognosis of EC patients and can be used as a new molecular marker to predict the survival rate of EC prognosis. hsa-mir-934, as an intronic miRNA of Vestigial-like 1, was found mainly expressed in BRCA1-associated triple-negative basal-like and sporadic breast cancer [45]. Another study also shows has-mir-934 to be significantly deregulated in H. pylori-induced gastric cancer [46]. This indicates that it may participate in the occurrence of EC by affecting the metabolic process of cancer cells. has-mir-940 can promote the proliferation, invasion, and metastasis of cancer cells by regulating the expression of target genes [47,48,49]. This study found that overexpression of hsa-mir-940 was associated with poor prognosis and reduced survival in endometrial cancer. hsa-mir-4758 participates in the post-transcriptional regulation of gene expression in multicellular organisms by affecting mRNA stability and translation, Wu et al. obtained the same results as this paper [50]. hsa-mir-4758 may serve as a novel biomarker for predicting survival in endometrial cancer. hsa-mir-146a is involved in posttranscriptional immune regulation of gene expression in multicellular organisms by affecting mRNA stability and translation. Some of the targets of its encoded miRNAs are tumor necrosis factor, interleukin-1 receptor-associated kinase 1, leukocyte Transcripts of interleukin 1-β, TNF receptor-related factor 6, and complement factor H. Thus, low expression of hsa-mir-146a can cause tumor cells to be unrecognized causing immune evasion and reducing inflammatory responses [51]. Both Shen et al. and Pastrello et al. have found hasmir-146a association with the early onset of familial breast and ovarian cancer and the passible mechanism is through BRCA1/2 mRNA expression regulation [52,53]. Low-expression of hsa-mir-3170 often leads to translational inhibition or instability of target mRNA, inducing tumor cell generation. In previous studies, hsa-mir-146a and hsa-mir-3170 have been identified as being associated with uterine endometrial carcinogenesis and progression, and as independent predictors of overall survival in uterine endometrial cancer patients [50]. hsa-mir-3614 can directly target upstream genes to promote cancer cell proliferation and invasion, and its role in renal clear cell carcinoma and non-small-cell carcinoma has been confirmed [51,52]. Similar to our study, Lu et al. also pointed out that has-mir-3614 is low-expressed in endometrial cancerous tissue [53]. A previous study had showed that the low-expression of has-mir-3616 contributes to triple negative breast cancer migration and invasion [54], and at the same time, low-expression of hsamir-3616 was also found to significantly reduce the survival rate of EC patients in this study (*p* < 0.001), which may be related to the expression of target mRNA. Translational inhibition or destabilization causes immunosuppression that forms the tumor microenvironment, and this can further be explored as potential targets of EC-related miRNAs. hsa-mir-4687 was identified as playing a role in lung adenocarcinoma [55] and the progression of colorectal cancer [56], and this study indicated that hsa-mir-4687 in the circulatory system of cancer patients was normal. The super channel of the hsa-mir-4687 gene includes class I MHC-mediated antigen processing, presentation, and antigen-activated B-cell receptor (BCR), resulting in the production of second messengers which can keep the body at homeostasis. The low expression of the hsa-mir-4687 gene leads to a decrease in the reactivity of the immune response, the formation of the tumor microenvironment and the occurrence of immune escape. The survival analysis of this study showed that the low-expression of hsa-mir-876 and hsa-mir-1269a had no significant effect on the survival rate of EC patients. Nevertheless, they were still differentially expressed in normal paracancerous tissues which is included in the construction of a nomogram model. In addition, the study by Wu et al. pointed out that hsa-mir-876 can be used as one of the indicators to predict the survival rate of endometrial cancer [50]. The down-regulation of hsa-mir-1269a has also been shown to play a role in other cancers [57,58]. Expanding the sample size can further explore the impact of these two miRNA molecular markers on the prognosis of endometrial cancer. “Omics science”, as a method to explore the molecular mechanism of cancer, combined with multidisciplinary, has been widely used to study the occurrence, treatment, and prognosis of gynecological diseases, including ovarian cancer [59,60], uterine endometriosis [61], and endometrial cancer [62]. In our study, 12 miRNAs were involved in constructing a nomogram model for predicting the survival of EC patients. Calibration curves for 3-year and 5-year survival probabilities showed that predictions using the nomogram model are in good agreement with those observed in the actual validation cohort, and ROC analysis showed that the risk prediction model has a good effect on assessing the prognosis of EC patients.

In conclusion, our study constructed a nomogram model to determine the prognosis of EC patients based on 12 miRNA variables, which is an independent prognostic factor of EC patients, and it can predict the survival of EC patients more accurately. In addition, the survival rate can be intuitively evaluated by dividing EC patients into high- and low-risk groups. This suggests that the expression of miRNA small molecule variables is an independent prognostic factor, which has a huge impact on patients. In addition, according to the survival analysis, hsa-mir138-2, hsa-mir-548f-1, hsa-mir-934, hsa-mir-940, and hsa-mir-4758, which are highly expressed in EC tissues, and hsa-mir-146a, hsa-mir-3170, hsa-mir-3614, hsa-mir-3616, and hsa-mir-4687, which are slightly expressed in EC tissues, are associated with poor prognosis and lower survival rate in endometrial cancer. Nevertheless, there are still some limitations to this study. We obtained the results of this study based on bioinformatics analysis. Therefore, further investigations are needed to verify the results of this study and determine the clinical value of these potential biomarkers for EC. It is very important to study the underlying mechanisms related to the occurrence and prognosis of endometrial cancer, which may provide a new direction for the future targeted therapy for endometrial cancer patients, determine the efficiency of the treatment, and predict the survival rate.

## Data Availability

The raw data of this study are obtained from the TCGA data portal (http://tcga.cancer.gov, accessed on 23 April 2022), which is a publicly available database.
